# Optimizing the Scope–Sheath Compatibility in RIRS: Matching of Reusable and Single-Use Flexible Ureteroscopes with FANS

**DOI:** 10.3390/jcm14207215

**Published:** 2025-10-13

**Authors:** Petrisor Geavlete, Razvan Multescu, Cosmin Ene, Bogdan Buzescu, Bogdan Geavlete

**Affiliations:** 1 Department of Urology, “Saint John” Emergency Clinical Hospital, 042122 Bucharest, Romania; geavlete@gmail.com (P.G.); bogdan_geavlete@yahoo.com (B.G.); 2 Faculty of General Medicine, “Carol Davila” University of Medicine and Pharmacy, 020021 Bucharest, Romania

**Keywords:** flexible ureteroscopy, reusable flexible ureteroscopes, single-use flexible ureteroscopes, FANS, ureteral access sheath

## Abstract

**Background/Objectives**: Adoption of single-use ureteroscopes (SU) and flexible and navigable suction ureteral access sheaths (FANS) have improved flexible ureteroscopy (fURS) efficiency and safety. However, the impact of scope–sheath pairing is less studied. This study aims to compare four scope–sheath configurations using reusable ureteroscopes (RU) and SU with either 11/13Fr or 12/14Fr FANS. **Methods**: We retrospectively evaluated 184 patients undergoing fURS for kidney solitary stones of 10–25 mm. Patients were manually matched across four groups: RU-11/13FANS, RU-12/14FANS, SU-11/13FANS, and SU-12/14FANS (46 patients in each). The endpoints were 30-day stone-free rate (SFR), operative time, surgeon-reported visibility (image clarity and procedural continuity) and postoperative complications. **Results**: Operative time was significantly shorter in single-use scope groups (*p* < 0.001). Visibility scores were highest in SU-12/14FANS and lowest in RU-11/13FANS across all subdomains. SFR was higher in SU groups. SU-11/13FANS had a significantly higher SFR than RU-12/14FANS (*p* = 0.027). In the reusable group, the use of 12/14Fr FANS was associated with a lower SFR compared to the 11/13Fr configuration. Complication rates were low (8.2% overall) and comparable among groups. **Conclusions**: Pairing SU with 12/14Fr FANS provided optimal visibility and good stone clearance without increasing complications. In contrast, RU paired with 12/14Fr FANS showed slightly reduced efficacy, possibly due to impaired maneuverability. The scope–sheath interaction influenced outcomes differently across scope types, underlining the importance of their matching in fURS.

## 1. Introduction

Flexible ureteroscopy has become an essential treatment alternative for renal calculi. Technological progress in digital visualisation systems, lasers and accessory instruments has significantly improved the efficacy and safety of the procedure. In recent years, single-use flexible ureteroscopes have become a viable and reliable alternative to reusable digital scopes, offering advantages related to sterility, cost predictability and consistent optical and mechanical performance [1,2].

The development of FANS has been associated with improved irrigation, efficient evacuation of debris and fragments, and reduced intrarenal pressure and septic complications [3,4]. While both the ureteroscope and ureteral access sheath individually influence surgical efficacy, there is limited evidence regarding their combined impact, particularly in terms of dimensional compatibility. Specifically, no prior study has systematically evaluated how pairing reusable and single-use flexible ureteroscopes with different FANS diameters affects operative performance, stone-free rates and procedural safety. The interaction between flexible ureteroscope types and sheath caliber may have practical consequences for procedural outcomes.

The present study aims to address this knowledge gap by comparing four distinct scope–sheath configurations: reusable ureteroscopes paired with either 11/13 Fr or 12/14 Fr FANS and small-diameter single-use ureteroscopes paired with the same set of sheath sizes. By directly assessing the clinical and technical implications of these pairings, we aim to identify which combinations best balance maneuverability, suction efficiency and safety in routine practice.

## 2. Materials and Methods

We performed a single-center retrospective study evaluating the medical records of adult patients who underwent flexible ureteroscopy for solitary stones between January 2024 and March 2025.

Patients aged 18 to 75 years with kidney solitary stones measuring 10–25 mm, confirmed on non-contrast computed tomography (CT), were included. Exclusion criteria were bilateral procedures, anatomical abnormalities (e.g., horseshoe kidney, calyceal diverticulum), history of urinary diversion, active urinary tract infection at the time of surgery, or incomplete clinical records.

All procedures were performed by one of two senior endourologists, each with extensive experience including more than 4000 RIRS cases. To ensure consistency, both surgeons agreed in advance on the interpretation of each visibility parameter. Surgeon identity was included in statistical analysis to assess the potential inter-operator variability.

Patients were allocated into four groups based on the type of used ureteroscope (Table 1) and the FANS size:

- Group RU-11/13FANS: Reusable 8.4 Fr flexible ureteroscope (Olympus URF-V2, Olympus Corporation, Hamburg, Germany) with 11/13 Fr FANS (ClearPetra, Well Lead Medical Co, Guangzhou, China);
- Group RU-12/14FANS: Reusable 8.4 Fr flexible ureteroscope (Olympus URF-V2, Olympus Corporation, Hamburg, Germany) with 12/14 Fr FANS (ClearPetra, Well Lead Medical Co, Guangzhou, China);
- Group SU-11/13FANS: Single-use 7.5 Fr flexible ureteroscope (Pusen 3033A, Pusen Medical, Zhuhai, China) with 11/13 Fr FANS (ClearPetra, Well Lead Medical Co, Guangzhou, China);
- Group SU-12/14FANS: Single-use 7.5 Fr flexible ureteroscope (Pusen 3033A, Pusen Medical, Zhuhai, China) with 12/14 Fr FANS (ClearPetra, Well Lead Medical Co., Guangzhou, China).

**Figure 1:**
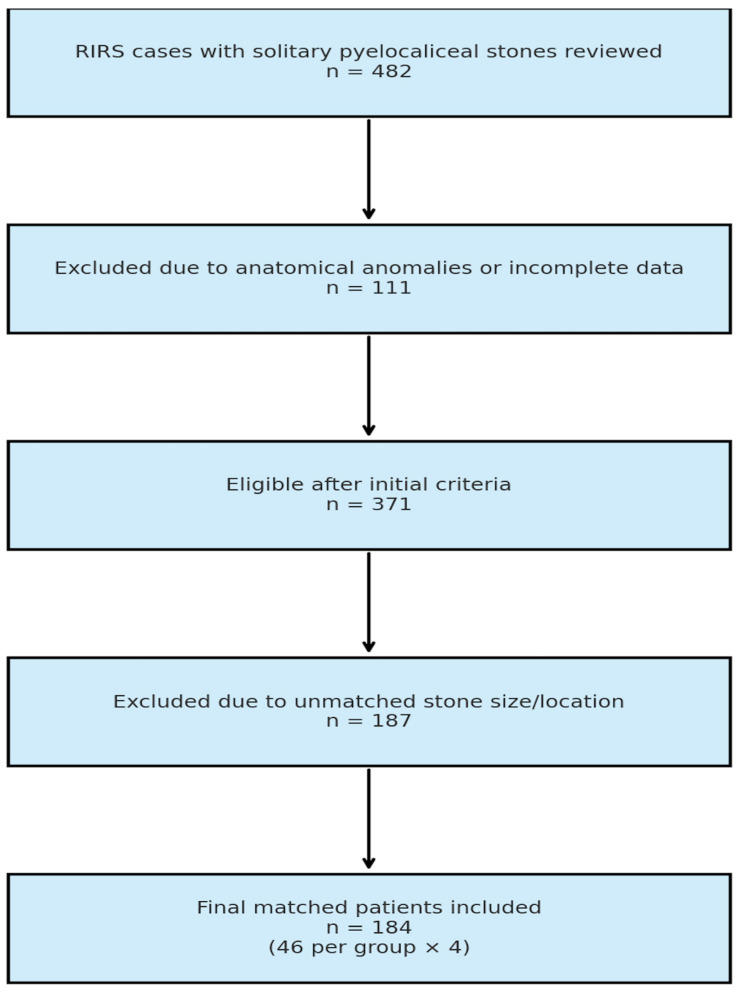
Selection process of the patients included in the study.

A total of 482 cases with single pyelocaliceal stones were reviewed, and after applying the initial inclusion and exclusion criteria, 371 patients remained eligible. From this cohort, 187 additional patients were excluded due to unmatched stone size and location, resulting in 184 patients selected via 1:1:1:1 manual matching. These were evenly distributed in the four study groups (*n* = 46 per group) (Figure 1).

To minimize selection bias, patients were manually matched in a 1:1:1:1 ratio across the four study groups based on two clinically relevant variables: stone size, matched within a ±2 mm tolerance and stone location, categorized as lower pole vs. non-lower pole. Only patients with complete medical records and full imaging data were eligible. All patients included in the study were pre-stented prior to surgery. Additional demographic and clinical variables such as age, sex, and ASA score were not used as matching criteria but were recorded for descriptive purposes.

Preoperative imaging with non-contrast CT was used to assess stone size, location, and density. Midstream urine culture was obtained in all patients. Those with positive cultures received culture-specific antibiotic therapy until microbiological resolution. A single dose of prophylactic antibiotic was administered perioperatively in culture-negative cases.

All interventions were performed under spinal anesthesia with the patient in lithotomy position. After the extraction of the JJ stent and an initial assessment of the ureter using a semirigid ureteroscope, a 0.035-inch hydrophilic guidewire was introduced into the pyelocaliceal system.

The FANS sheath, either the 11/13 Fr or 12/14 Fr, was advanced over the guidewire under fluoroscopic control. The flexible ureteroscope was inserted, and laser lithotripsy was performed using a 70 Watts MegaPulse 70+ Holmium:YAG laser (Richard Wolf, Knittlingen, Germany). Gravity irrigation with room temperature saline bags suspended at 50 cm above the patient was modulated manually. The sheath’s suction port was connected to a vacuum source, with negative pressure set between −20 and −40 kPa. A double-J ureteral stent was routinely placed at the end of the procedure.

KUB radiography was obtained on the first postoperative day. Stents were removed after 3 to 4 weeks, and a non-contrast CT scan was performed at 30 days to assess residual fragments. Stone-free status was defined as the absence of any fragment ≥ 2 mm.

The primary endpoint was the 30-day SFR. Secondary endpoints included operative time (from start of laser lithotripsy until flexible ureteroscope removal from the pyelocaliceal system), complication rates (graded based on the Clavien-Dindo classification), and surgeon-reported assessments of visibility.

All reusable ureteroscopes were routinely serviced and maintained according to manufacturer recommendations and institutional standards to ensure optimal performance during the study period.

Visibility scores were prospectively recorded at the time of surgery as part of the institutional quality monitoring program and previously approved observational studies evaluating flexible ureteroscopes and FANS performance. For the present study, we retrospectively analyzed this pre-existing data, focusing on scope–sheath pairing. All scores were documented by the operating surgeon immediately after the procedure using the same standardized 5-point Likert scale. At the time of their recording, the surgeons were unaware that these scores would be later analyzed in relation to scope–sheath matching, thus reducing the risk of bias. Intraoperative visibility was assessed by the operating surgeon immediately after each procedure, by rating two specific parameters. The first one was image clarity during laser lithotripsy (Clarity Score, rated using a 5-point Likert scale from 1 = very bad to 5 = excellent). The second one was the need to pause the procedure due to poor visibility (Continuity Score, rated also using a Likert scale from 1 = very often to 5 = very rare). The sum of the two scores (Composite Visibility Score) was also compared.

Statistical analyses were performed using SPSS version 26.0 (IBM Corp., Armonk, NY, USA). Continuous variables were reported as mean ± standard deviation and analyzed using two-way analysis of variance (ANOVA) to evaluate the effects of ureteroscope type, FANS sheath size, and their interaction. Categorical variables were compared using the chi-square test. Multivariable logistic regression was used to adjust for residual confounding. A *p*-value < 0.05 was considered statistically significant.

## 3. Results

A total of 184 patients were included, equally distributed in the four study groups (46 patients in each group). Baseline characteristics, including age, sex and ASA scores were comparable between groups, with no statistically significant differences. Stone size, location (lower pole vs. non-lower pole) and composition were matched in the selection process (Table 2). The mean stone volume across the cohort was approximately 1824 ± 76 mm^3^.

The mean operative time varied significantly across groups (Table 3, *p* < 0.001). The shortest mean operative time was observed in the SU-11/13FANS group (41.1 ± 3.1 min), followed by SU-12/14FANS (44.0 ± 3.9 min). Reusable scope groups had similar operative times: 53.3 ± 3.1 min for the RU-12/14FANS and 53.8 ± 3.9 min for RU-11/13FANS.

**Figure 2:**
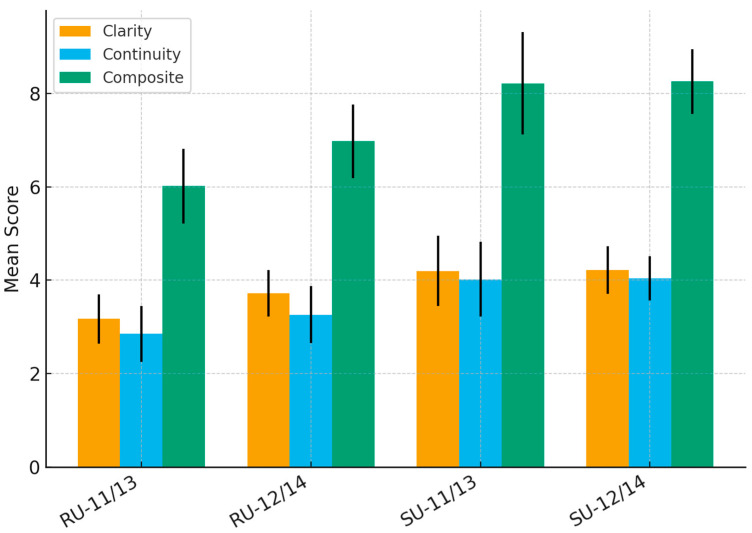
Visibility outcomes (clarity, continuity, composite scores) across the four groups. Black bars represent standard deviation.

**Figure 3:**
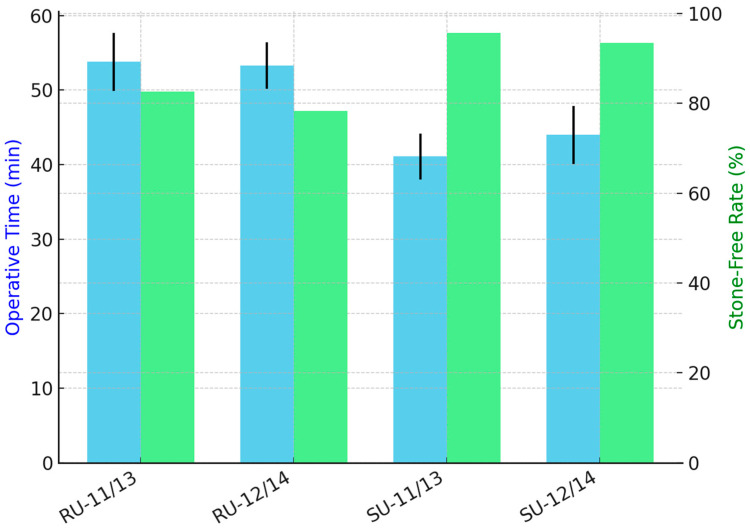
Operative time (minutes, blue bars) and stone-free rate (%, green bars) across the four groups. Black bars represent standard deviation for operative time.

Surgeon-reported visibility scores are presented in Table 3 and Figure 2 and Figure 3. Image Clarity Score was highest in SU-12/14FANS (4.29 ± 0.51) followed by SU-11/13FANS (not statistically significant), and lowest in RU-11/13FANS (3.17 ± 0.53). Continuity Score was highest in SU-12/14FANS (4.04 ± 0.47) and lowest in RU-11/13FANS (2.85 ± 0.60). Composite Visibility Score followed a similar distribution: 8.33 ± 0.69 for SU-12/14FANS, 8.21 ± 0.99 for SU-11/13FANS, 6.98 ± 0.79 for RU-12/14FANS, 6.02 ± 0.80 for RU-11/13FANS.

For descriptive purposes, visibility scores were also grouped into categories (low, moderate, high). This classification was used only to facilitate interpretation for readers, while all statistical analyses were performed using the original continuous Likert scale values. As shown in Table 4, these categories mirrored the statistical findings: both reusable groups clustered in the low/moderate range, both single-use groups in the high range.

At 30-day follow-up, stone-free status was achieved in 78.3% (36/46 patients) of RU-12/14FANS group, 82.6% (38/46 patients) of RU-11/13FANS group, 95.7% (44/46 patients) of SU-11/13FANS group and 93.5% (43/46 patients) of SU-12/14FANS group. Regarding the stone-free rate, the overall comparison using Chi-square analysis demonstrated a statistically significant difference among the four groups (*p* = 0.031).

Pairwise analysis showed that SU-11/13FANS had a significantly higher stone-free rate than RU-12/14FANS (Fisher’s *p* = 0.027; Chi-square test *p* = 0.030). All other comparisons were non-significant (*p* > 0.09).

Complications occurred in 15 of 184 patients (8.2%), with no significant difference between the four study groups (Chi-square test *p* = 0.850). The rates were 8.7% in RU-11/13FANS, 10.9% in RU-12/14FANS, and 6.5% in both SU-11/13FANS and SU-12/14FANS. Most complications were minor (Clavien I) and resolved with conservative treatment. One Clavien II (pain requiring medication) and one Clavien IIIa event (stent repositioning) were recorded, both in the RU-11/13FANS group. No Clavien IV or V events occurred.

Use of single-use ureteroscope was associated with reduced operative time (*p* < 0.01). Use of 12/14 FANS was associated with higher visibility scores (*p* < 0.01). The combination SU-11/13FANS had the strongest association with achieving SFR (*p* = 0.007).

Detailed pairwise statistical comparisons for visibility scores, operative time, and stone-free rates are provided in Supplementary Materials Table S1.

## 4. Discussion

Evaluation of practice patterns demonstrated that most endourologists do not exclusively use single-use flexible ureteroscopes, but routinely mix reusable and disposable models, particularly in high-volume tertiary centers [5,6,7]. Therefore, identifying the optimal scope-sheath combinations for different situations is important. Our study aimed to assess how different pairings of reusable or single-use flexible ureteroscopes and FANS may influence postoperative stone-free rates.

All four scope–sheath configurations yielded high overall stone-free rates, confirming the conclusions of previously published articles evaluating the impact of FANS on the efficacy of the interventions. Various studies already demonstrated that procedures performed with suction sheaths have higher stone-free rates and a reduced need for reinterventions by comparison to standard sheaths [8].

However, our comparison revealed statistically significant differences in intraoperative parameters, suggesting that the interaction between scope type and sheath diameter may influence clinical outcomes.

Single-use ureteroscopes offer several certain advantages compared to reusable ones: there is no need for resterilization with a potential effect on cross infections, they offer optimal mechanical and optical performance at every procedure, they can be used by beginners without increasing the costs, and they can be used with various accessory instruments or in challenging situations (lower pole stones etc.) without the fear of damaging the scope. A meta-analysis comparing surgical outcomes in patients who underwent flexible ureteroscopy procedures using single-use and reusable ureteroscopes reported no statistically significant differences in operating time, stone-free rates, complication rates or hospital stay length [9].

Pairing the flexible ureteroscope with FANS is a possible game changer, with the potential to reshape the way the flexible ureteroscopy approach is performed. Various parameters may influence this combination: diameter and flexibility of the sheath, amount of space between the sheath wall and the ureteroscope, ability to place it in the calyx, the area where lithotripsy is performed, etc. [10].

Regarding the operative time, it differed significantly between various scope–sheath configurations, with the shortest durations recorded for single-use ureteroscopes paired with both 11/13 Fr and 12/14 Fr FANS. The mean time savings compared to reusable scope groups exceeded 9 min, which is both statistically and clinically relevant. Several factors may account for this difference. First, the reduced shaft diameter of single-use ureteroscopes improves irrigation flow when combined with FANS, allowing faster fragments clearance and maintaining good visibility. Second, the consistently high optical performance of new, disposable ureteroscopes eliminates the variability in image quality and maneuverability sometimes associated with reusable instruments after repeated reprocessing and repairs. The combination of good visibility, access, and efficient fragment evacuation likely reduces the number of pauses during lithotripsy, thereby shortening the procedure.

Interestingly, sheath size did not produce a significant difference between the two single-use groups, suggesting that for these thin scopes, the irrigation–suction dynamics may already be almost optimal with the smaller FANS diameter. In contrast, reusable scopes, having larger outer diameters, showed similar longer operative times regardless of sheath size, likely because the relative clearance between the scope and sheath was insufficient to offer substantial differences in irrigation efficiency.

From a clinical standpoint, the observed reduction in operative time with single-use devices paired with smaller caliber FANS could contribute to lower intrarenal pressures, reduced anesthesia exposure, and improved operating room efficiency, without compromising procedural safety.

In our study, patients treated with single-use ureteroscopes paired with 11/13 Fr FANS achieved the highest SFR (95.7%), which was significantly higher than that observed in the group that underwent reusable ureteroscope with 12/14 Fr FANS (78.3%). This difference remained statistically significant across both Fisher’s exact test and Chi-square analysis. No other pairwise comparison reached significance, but a consistent trend favoring single-use scopes was observed across multiple analyses.

Interestingly, within the reusable scope group, the 11/13 Fr sheath achieved a higher SFR than the 12/14 Fr sheath, despite the latter’s theoretical advantage in fragment evacuation and visibility. This counterintuitive finding may reflect the greater flexibility of thinner sheaths, which may improve ergonomic access to calyces, particularly in anatomically challenging positions such as the lower pole which may have improved stone clearance. Larger sheaths, though potentially more effective for suction and debris evacuation, may be limited by ureteral tortuosity, restricted advancement, or because the clearance between the 8.4 Fr scope and the sheath remained relatively small, ultimately compromising access to stone and clearance. These findings highlight that sheath size does not uniformly predict clinical outcomes and that ergonomics and anatomical access also play a decisive role.

In contrast, single-use scopes achieved high stone-free rates regardless of sheath size, suggesting that their smaller shaft diameter and improved deflection characteristics may compensate for limitations in sheath flexibility. The lack of difference between 11/13 and 12/14 Fr FANS in the single-use group supports this interpretation, implying that scope properties may play a dominant role in this context.

These findings highlight the importance of considering not only the individual attributes of ureteroscopes and access sheaths but also their functional compatibility. Optimizing the scope–sheath pairing may be particularly critical in complex cases where stone location or anatomy limits maneuverability. Further prospective studies are warranted to validate these observations and explore whether tunning scope–sheath combinations to anatomical scenarios can further improve RIRS outcomes.

The literature reports various complication rates associated with flexible ureteroscopy approach. Most of them are mild but quite heterogeneous among different studies [11,12].

Use of suction ureteral access sheaths seems to be associated with a smaller complication rate. Zhu et al. found that the overall complication rate was only 11.5%, significantly lower than the rate of 24.8% recorded in patients in which the standard sheaths were used [13]. When discussing FANS specifically, a meta-analysis evaluating 8 studies (one randomized controlled trial and 7 observational ones) found significant lower overall complication rates and also reduced risk of septic complications when suction accessories were used by comparison to standard ones [14]. The findings in our study, which employs FANS in all evaluated patients, are consistent with literature, showing a reduced rate of complications of only 8.2%. Almost all recorded problems are mild, and with a similar incidence among the four groups.

We observed that the RU-12/14FANS and SU-11/13FANS pairings, which theoretically provide a similar space between the scope and the sheath, and thus a similar clearance, produced different outcomes. Although speculative, there are some possible explanations for this observation. Single-use devices provide consistent optical quality and deflection at each procedure, whereas mechanical wear in reusable devices may influence handling despite routine servicing and maintenance. Furthermore, a previous study from our group demonstrated that single-use scopes, despite having the same 3.6 Fr working channel, allow higher irrigation flow compared with reusable scopes [15]. This may likely enhance intraoperative visibility, facilitate fragment evacuation and may contribute to the superior operative efficacy observed with SU-11/13FANS.

While the optical performance of reusable ureteroscopes is generally stable over repeated use, mechanical parameters including deflection may progressively decline despite routine servicing [16]. This reflects real-world conditions of reusable devices and contrasts with single-use scopes, which provide consistent optical and mechanical performance in every procedure. As mentioned before, our reusable ureteroscopes were not brand new at the time of their use but routinely serviced and maintained according to manufacturer recommendations and institutional standards. We consider this apparent drawback highly relevant as it represents an inherent aspect of clinical practice and should be taken into account when comparing reusable and single-use ureteroscopes.

This study also has several limitations that should be acknowledged. It was conducted in a single high-volume tertiary referral center, which may limit the generalizability of the results to institutions with different case volumes, equipment availability or surgical expertise. Although our manual matching approach aimed to reduce selection bias, the retrospective design inherently carries this risk. The sample size, while balanced across the four groups, remains relatively modest, especially for detecting smaller differences in secondary endpoints such as complications. All procedures were performed by two highly experienced urologists, which minimizes operator-related variability but may not reflect the results in lower-volume centers or among less experienced surgeons. Finally, we did not include a cost-effectiveness analysis, which is particularly relevant in the context of hybrid use of reusable and single-use ureteroscopes.

## 5. Conclusions

In our matched analysis, single-use flexible ureteroscopes paired with 11/13 Fr FANS achieved the shortest operative times and highest stone-free rate, significantly outperforming reusable ureteroscopes with 12/14 Fr sheaths. Sheath size had no significant effect within the single-use group, while in reusable scopes, the smaller 11/13 Fr sheath produced numerically better outcomes. In high-volume centers that employ both reusable and single-use flexible ureteroscopes, our findings may optimize pairing strategies with FANS. Single-use scopes with 11/13 Fr FANS may be the optimal choice when procedural efficiency and complete clearance are priorities. In reusable scopes, sheath size did not meaningfully influence outcomes, suggesting that anatomical factors and surgeon preference may guide this selection. These results support a tailored approach to maximize clinical benefit.

## Figures and Tables

**Table 1:** Technical characteristics of flexible ureteroscope models used in the study.

	Reusable (RU)—Olympus URF-V2	Single-Use (SU)—Pusen 3033A
Shaft diameter	8.4 Fr	7.5 Fr
Working channel	3.6 Fr	3.6 Fr
Tip deflection	270° (up/down)	270° (up/down)
Field of view	90°	90°
Imaging system	Digital	Digital
Reusability	Reusable, routinely serviced	Single-use, disposed after each case

**Table 2:** Baseline characteristics of the study groups.

Variable	RU-11/13 (*n* = 46)	RU-12/14 (*n* = 46)	SU-11/13 (*n* = 46)	SU-12/14 (*n* = 46)	*p*-Value
Age (years), mean ± SD	50.2 ± 10.1	51.1 ± 9.8	49.6 ± 11.2	49.7 ± 10.5	ns
Male sex, *n* (%)	30 (65.2%)	32 (69.6%)	29 (63%)	31 (67.4%)	ns
ASA score ≥ 3, *n* (%)	11 (23.9%)	11 (23.9%)	10 (21.7%)	11 (23.9%)	ns
Stone volume (mm^3^), mean ± SD	1792 ± 56	1836 ± 81	1864 ± 62	1804 ± 78	matched
Lower pole stones, number (%)	20 (43.5%)	20 (43.5%)	20 (43.5%)	20 (43.5%)	matched
Stone composition (CaOx/UA/Other), number	26/15/5	26/15/5	26/15/5	26/15/5	matched

ns: not significant.; SD: standard deviation.

**Table 3:** Operative times, Clarity Scores and Continuity Scores recorded in the four study groups.

Group	Mean Clarity Score	Clarity Score SD	Mean Continuity Score	Continuity Score SD	Mean Operating Time (min)	Operating Time SD
RU-11/13FANS	3.17	0.53	2.85	0.60	53.8	3.9
RU-12/14FANS	3.72	0.50	3.26	0.61	53.3	3.1
SU-11/13FANS	4.20	0.75	4.02	0.80	41.1	3.1
SU-12/14FANS	4.29	0.51	4.04	0.47	44.0	3.9

SD: standard deviation.

**Table 4:** Comparison of the outcomes of visibility scores in the four study groups.

Group	Mean Clarity Score	Mean Continuity Score	Mean Composite Score	Comments
RU-11/13FANS	Low	Very Low	Lowest	Worst performer consistently
RU-12/14FANS	Moderate	Improved	Moderate	Gains in both clarity and continuity
SU-11/13FANS	High	High	High	Excellent in both metrics
SU-12/14FANS	High	High	High	Slight edge in clarity but not continuity

## Data Availability

The original contributions presented in this study are included in the article. Further inquiries can be directed to the corresponding author.

## Supplementary Materials

The following supporting information can be downloaded at: https://www.mdpi.com/article/10.3390/jcm14207215/s1, Table S1: Pairwise statistical comparisons between study groups for visibility scores, composite score, operative time, and stone-free rate. Results are expressed as *p*-values from post-hoc tests; *ns* = not significant.

## Abbreviations

The following abbreviations are used in this manuscript:

ASA	American Society of Anesthesiologists
CaOx	Calcium oxalate
FANS	Flexible and navigable suction ureteral access sheaths
fURS	Flexible ureteroscopy
RIRS	Retrograde intrarenal surgery
RU	Reusable ureteroscope
SD	Standard deviation
SFR	Stone-free rate
SU	Single-use ureteroscope
UA	Uric acid
