# Supplementary material for: Optimizing the Scope–Sheath Compatibility in RIRS: Matching of Reusable and Single-Use Flexible Ureteroscopes with FANS
Source: J Clin Med. 2025 Oct 13;14(20):7215. doi: 10.3390/jcm14207215 (PMC12564809; doi:10.3390/jcm14207215)
Supplement: Supplementary file 1 [file jcm-14-07215-s001.zip › jcm-3858088-supplementary.pdf]

Comparison	Clarity Score	Continuity Score	Composite Score	Operative Time	Stone-Free Rate
RU–11/13 vs RU–12/14	ns	ns	$p < 0.01$	ns	ns
SU–11/13 vs SU–12/14	ns	ns	ns	ns	ns
RU–11/13 vs SU–11/13	$p < 0.001$	$p < 0.001$	$p < 0.001$	$p < 0.001$	ns
RU–12/14 vs SU–11/13	$p < 0.001$	$p < 0.001$	$p < 0.001$	$p < 0.001$	p=0.027 (Fisher); 0.030 (Chi-square)
RU–12/14 vs SU–12/14	$p < 0.001$	$p < 0.001$	$p < 0.001$	$p < 0.001$	ns

Supplementary Table S1: Pairwise statistical comparisons between study groups for visibility scores, composite score, operative time, and stone-free rate. Results are expressed as p-values from post-hoc tests; *ns* = not significant.
